# Valorization of *Citrus reticulata* Blanco Peels to Produce Enriched Wheat Bread: Phenolic Bioaccessibility and Antioxidant Potential

**DOI:** 10.3390/antiox12091742

**Published:** 2023-09-08

**Authors:** Esther Gómez-Mejía, Iván Sacristán, Noelia Rosales-Conrado, María Eugenia León-González, Yolanda Madrid

**Affiliations:** Department of Analytical Chemistry, Faculty of Chemistry, Complutense University of Madrid, 28040 Madrid, Spain; ivasacri@ucm.es (I.S.); leongon@ucm.es (M.E.L.-G.); ymadrid@ucm.es (Y.M.)

**Keywords:** mandarin peels, polyphenols, enriched wheat bread, waste valorization, in vitro bioaccessibility, antioxidant activity

## Abstract

The fortification of foods with bioactive polyphenols aims to improve their functional properties and to provide health benefits. Yet, to exert their benefits, phenolic compounds must be released from the food matrix and absorbed by the small intestine after digestion, so assessing their bioaccessibility is crucial to determine their potential role. This work aims to incorporate *Citrus reticulata* Blanco peel extracts into wheat bread as a promising opportunity to increase their bioactive potential, along with supporting the sustainable management of citrus-industry waste. A control and a wheat bread enriched at 2% and 4% (*w*/*v*) with a phenolic extract from mandarin peels were prepared and analyzed for antioxidant activity and phenolic composition using LC-MS and UV-Vis spectrophotometry. In addition, in vitro digestion was performed, and the digested extracts were analyzed with HPLC-MS/MS. The results showed a significant increase in total flavonoid content (TFC, 2.2 ± 0.1 mg·g^−1^), antioxidant activity (IC_50_ = 37 ± 4 mg·g^−1^), and contents of quercetin, caffeic acid, and hesperidin in the 4% (*w*/*v*) enriched bread. Yet, most polyphenols were completely degraded after the in vitro digestion process, barring hesperidin (159 ± 36 μg·g^−1^), highlighting the contribution of citrus enrichment in the development of an enriched bread with antioxidant potential.

## 1. Introduction

Over the last decade, consumers’ nutritional awareness has increased considerably around the world, leading to the search for healthier and more nutritious foods that ensure greater safety and promote well-being [1]. As a result, a growing interest is now being shown in foods that, in addition to providing basic nutrients, contain a high antioxidant potential [2]. According to scientific reports, the deficiency of antioxidants in the daily diet is not only the cause of the organism aging, but also of the appearance of numerous diseases of affluence derived from oxidative stress [2], including cancer, Alzheimer’s disease, obesity, and osteoporosis, among others [3,4]. For this reason, both industries and researchers are engaged in optimizing food production technology to develop enriched foodstuffs with functional components, which improve the quality, taste, performance, and bioavailability of food products, whilst also reducing the burden on health services [1,3].

One of the most abundant families of natural antioxidants are phenolic compounds. Polyphenols are secondary metabolites synthesized by plants as essential physiological compounds, comprising a large family of molecules with one or more phenolic rings [4]. In addition to being potent antioxidants, they are also known to exhibit anti-allergic, antiviral, anti-inflammatory, and antimutagenic properties [3,4]. These compounds are present in many plant foods, such as citrus fruits. *Citrus* (Rutaceae) is the most widely produced tree fruit crop in the world, with more than 130 million tons per year. About 33% of citrus fruits are processed industrially for juice production, resulting in the generation of over 15 million tons of citrus waste per year, namely peels, membranes, and seeds, the management of which is a major economic and environmental concern [5]. These citrus by-products, and in particular the peel, are rich in bioactive polyphenols such as hesperidin, coumaric acid, ferulic acid, rutin, and luteolin, among others [6,7]. Therefore, based on their composition, their low cost, and their easy availability, citrus waste peels should be considered a potential nutraceutical source of antioxidant and value-added ingredients for the elaboration of functional foods. Therefore, this approach allows one to follow a circular economy strategy that offers alternative and green opportunities for the disposal of citrus waste, allowing for more sustainable production and consumption within the agri-food sector [5,6]. In general, cereal-based foods, such as bread and bakery products, play an important role in human nutrition, given that they are considered a good source of energy and irreplaceable nutrients. In fact, they represent up to 50% of dietary intake in some communities [2,3]. Thus, given their widespread consumption, bread and bakery products are considered to be the foremost appropriate carriers of functional supplements. Previously, Taglieri et al. studied the preparation of a bread enriched with 8% (*w*/*w*) purple potato flour and 0.75% (*w*/*w*) sour orange (*Citrus* × *aurantium*) albedo, drawing a significant rise in phenolic compounds such as apigenin 7-*O*-ruthinoside and neohesperidin, as well as a higher antioxidant activity and a longer shelf life of the enhanced bread [8]. On the other hand, Yaqoob et al. prepared cookies enriched with kinnow (*Citrus reticulate* L.) pomace and peel powder at 5–20% (*w*/*w*) and citrus peel phenolic extract at 1–4% (*w*/*w*). As a result, a significant improvement in the DPPH antioxidant capacity as well as in the total contents of phenolic compounds, flavonoids, and carotenoids was detected when the cookies were enriched with the phenolic extracts. In addition, a greater oxidative stability of the citrus-residue-enriched cookies was noted compared with those with the artificial antioxidant butylated hydroxyanisole [9]. And Laganà et al. substituted 2.5% to 15% (*w*/*w*) wheat flour with bergamot (*Citrus bergamia*) pomace to obtain improved cookies, showing a proportional increase in total phenolic content and total flavonoid content in the enriched cookies, especially at the highest level of enrichment (3.64 mg GAE·g^−1^ and 3.90 mg CE·g^−1^, respectively) [10]. Although, in the aforementioned studies, the preparation of fortified foods was carried out by adding the bioactive residue or extract in a solid state [8,9,10], a number of advantages regarding diffusion, dosage, and application are well-known when ready-to-use nutraceutical ingredients are available in a liquid state. Furthermore, solvents can provide synergistic activity not only in the overall extraction of polyphenolic compounds, but also in their underlying bioactivity [11]. Therefore, both facts seem to indicate that the formulation of natural additives in liquid form for the preparation of fortified foods is becoming increasingly attractive.

Ultimately, an issue to be taken into consideration when designing an enriched food is the stability of the bioactive compounds involved in the digestion process, which may influence not only their bioaccessibility but also their potential activity [12]. Since phenolic compounds are usually degraded by oxidation during digestion, it is essential to evaluate their digestive stability, generally using in vitro gastrointestinal procedures, in which the mechanical and physiological conditions of each stage of the gastrointestinal digestion process are mimicked [13].

In view of the above, this study focuses on exploring the feasibility of preparing wheat bread enriched with different ratios of hydroalcoholic phenolic extract derived from mandarin peels. The resulting breads were compared in terms of total phenolic and flavonoid content and DPPH antioxidant activity using spectrophotometric methods. Furthermore, the individual phenolic composition was studied and the in vitro digestive stability of low-molecular-weight polyphenols, i.e., phenolic acids, flavonoids, and stilbenes, in the prepared breads was evaluated using liquid chromatography-mass spectrometry.

## 2. Materials and Methods

### 2.1. Raw Citrus Material

‘Orri’ hybrid mandarins (*Citrus reticulata* Blanco) were purchased at a local market in Madrid (Spain). The peels were manually separated from the fruit, cleaned with Milli-Q water, and cut into equally sized portions (1 × 0.5 cm) using stainless-steel scissors. Finally, they were stored in hermetically sealed glass containers at −20 °C until processing.

### 2.2. Reagent, Solvent, and Polyphenol Standards

Analytical-grade reagents and purified water from a Milli-Q system (Merck, Madrid, Spain) were used. Absolute ethanol HPLC gradient quality (EtOH), acetonitrile (ACN), methanol (MeOH), and formic acid (FA) of MS quality were provided by Scharlab (Barcelona, Spain). Dimethyl sulfoxide (DMSO, ≥99.9%), 2N Folin–Ciocalteu reagent, 2,2-diphenyl-1-picrylhydrazyl (DPPH), urea (≥99.5%), α-amylase from Bacillus subtilis (powder, 50 U/mg), bile salts, pepsin from porcine gastric mucosa, and pancreatin from porcine pancreas were all supplied by Sigma-Aldrich (St. Louis, MO, USA). Ammonium molybdate tetrahydrate, aluminum chloride 6-hydrate, sulfuric acid (95–98%), sodium carbonate anhydrous, tri-sodium phosphate 12-hydrate, monosodium phosphate monohydrate (≥98.0%), anhydrous sodium sulphate (≥99.9%), potassium chloride (99.5%), potassium thiocyanate (98.0%), sodium bicarbonate (99.5%), hydrochloric acid (37%), sodium hydroxide, and sodium nitrite were obtained from Panreac (Barcelona, Spain).

Phenolic-standard gallic acid monohydrate (≥98.0%), dihydroxybenzoic acid (≥97.0%), chlorogenic acid (≥95.0%), catechin (≥98.0%), caffeic acid (≥98.0%), *p*-coumaric acid (≥98.0%), epicacatechin (≥98.0%), *trans*-ferulic acid (98%), rutin trihydrate (≥95.0%), myricetin (≥96.0%), resveratrol (≥99.0%), quercetin (≥95.0%), kaempferol (≥97.0%), and naringin (≥95.0%) were obtained from Sigma-Aldrich (St. Louis, MO, USA). Hesperidin (≥98.0%) was provided by European Pharmacopoeia. Phenolic stock solutions (200 mg·L^−1^) were prepared in MeOH, ethanol–water mixture 80:20 (*v*/*v*) (quercetin), or 5% (*v*/*v*) DMSO aqueous solution (hesperidin). They were stored in the dark at 4 °C or at −80 °C (myricetin, hesperidin, *trans*-ferulic, and caffeic acid) for up to one month. Fresh working standard solutions were prepared daily by diluting stock solutions as needed.

### 2.3. Moisture Determination

The moisture content of mandarin peel and bread samples was obtained according to AOAC method 20.013 [14]. Briefly, 1.0000 g of sample, previously ground, was weighed in triplicate and dried to constant weight at 105 °C (±0.1 °C) (P-Selecta oven, Panreac, Barcelona, Spain). The free-water content was calculated gravimetrically and expressed as a percentage (mean ± standard deviation, n = 3).

### 2.4. Citrus reticulata Blanco Peel Extract Preparation

Polyphenols from mandarin peels were extracted using a procedure previously optimized elsewhere [15], with slight modifications. Heat-assisted solid–liquid extraction (SLE) was performed at 90 °C under magnetic stirring (3 rpm) for 15 min (VELP Scientifica, Us-mate, MB, Italy) using 1.5000 g (2%, *w*/*v*) or 3.0000 g (4%, *w*/*v*) of citrus peel waste and 75 mL of 20:80 (*v*/*v*) ethanol–water. The obtained extracts were cooled to room temperature and centrifuged at 1528× *g* (centrifuge 5804, Eppendorf, Sigma Aldrich, Madrid, Spain). Finally, the supernatants were collected for further use and analysis. Extracts were prepared in triplicate.

### 2.5. Production of Enriched Wheat Bread

The enriched bread was prepared by mixing 125 g of T65-strength wheat flour, 1.7500 g of dry yeast, 0.2500 g of salt, and 3.7500 g of extra virgin olive oil (Picual variety) with 75 mL of 2% (*w*/*v*) or 4% (*w*/*v*) mandarin peel extract. Additionally, a control bread was prepared using a 20:80 (*v*/*v*) ethanol–water mixture. All ingredients were manually kneaded for 10 min until a homogeneous dough was formed, which subsequently fermented for 2 h at 27 °C. The dough was then divided into three portions of approximately 60 g and put into baking pans lined with parchment paper. Finally, control and enriched breads were baked at 200 °C for 20 min in a P-Selecta oven (Panreac).

### 2.6. Phenolic Extraction of Enriched Wheat Bread

An amount of 1.000 g of ground wheat bread was added to 20 mL of 70:30 (*v*/*v*) ethanol–water mixture and held at 60 °C under magnetic stirring (3 rpm) for 30 min. The phenolic extract was cooled to room temperature and centrifuged at 11,000 rpm for 10 min. Ultimately, the supernatant was collected for further analysis. Samples were prepared in triplicate.

The selection of the aforementioned procedure was based on a study in which the effect of the extraction technique on the extraction efficiency was evaluated using total phenolic content (TPC), total flavonoid content (TFC), and antioxidant activity determinations, using the control bread as a matrix model, while maintaining the extraction solvent (70:30 (*v*/*v*) ethanol–water) and the sample-to-solvent ratio (1/20). The extraction techniques assessed were SLE assisted by mechanical stirring (29.5 kHz, 25 °C, P-Selecta, Barcelona, Spain), SLE assisted by magnetic stirring and heating (3 rpm, 60 °C, VELP Scientifica, Us-mate, Italy) for 30 min, and ultrasound-assisted extraction (UAE) working at 40% of amplitude and 25 °C for 20 min. The UAE was performed using two different instruments, an ultrasonic homogenizer (HD 3200, Sonopuls, Bandelin, Berlin, Germany) equipped with a titanium probe (25 × 15.5 × 25.7, mm) fitted with a 200 W high-frequency generator at 20 kHz frequency, and a Vibra-Cell ultrasound probe (Sonic, Newton, CT, USA) equipped with a 2 mm diameter titanium microtip fitted with a 130 W high-frequency generator at 20 kHz frequency.

### 2.7. Spectrophotometric Analysis of Phenolic Extracts: Total Polyphenol Content, Total Flavonoid Content, and Antioxidant Activity

The total phenolic content (TPC) of mandarin peel and wheat bread extracts were assessed using the Folin–Ciocalteu method [16]. The phenolic extracts (1 mL for wheat bread or 400 µL for mandarin peel) were mixed with 70 µL of the Folin–Ciocalteu reagent and 60 µL of 7.5% (*w*/*v*) Na_2_CO_3_ to a final volume of 10 mL. Gallic acid was used as standard for external calibration (0–40 µM, n = 5). The absorbance of the solutions was measured at 720 nm (UV-Vis spectrophotometer Cary 60, Agilent Technologies, Madrid, Spain) and the results were expressed as mg of gallic acid equivalent per g of dry sample (mg GAE·g^−1^). The assay was performed in triplicate.

The total flavonoid content (TFC) was determined according to the aluminum complexation colorimetry assay [16]. Sample aliquots (1 mL for wheat bread or 500 µL for mandarin peel) were added to 2 mL of water, 150 µL of 5% (*w*/*v*) NaNO_2_, and 150 µL of 10/(*w*/*v*) AlCl_3_. After two incubation periods of 5 min, 1 mL of 1 M NaOH was mixed with the reaction solution and stored for 15 min; finally, the solution was diluted to 10 mL with water. The absorbance of the solutions was measured at 450 nm using a UV-Vis spectrophotometer. Quercetin was used, as is standard, to obtain a calibration curve (0–45 µM, n = 6) and the results were expressed as mg of quercetin equivalent per g of dry sample (mg QE·g^−1^). Samples were analyzed three times.

The antioxidant activity of wheat bread and mandarin peel samples was assessed based on the ability of the extracts to scavenge the DPPH radical [17]; thus, they were assessed in terms of their antiradical activity. Working solutions were prepared combining 500 µL of 0.28 mM DPPH (MeOH) and 100–500 µL of sample aliquots. In addition, a DPPH control and a blind control (sample aliquot plus pure MeOH) were prepared. After a 30 min incubation in the dark, the absorbance was measured at 515 nm. Antioxidant activity was tested in triplicate and denoted as IC_50_ value in mg of extract per g of dry sample.

### 2.8. Chromatographic Analysis of Phenolic Extracts Using cLC-ESI-MS and HPLC-ESI-QTOF

Individual polyphenol determination of mandarin and wheat bread extracts was carried out using capillary liquid chromatography coupled to a mass simple quadrupole analyzer (cLC-ESI-MS), following the procedure previously described by Gómez-Mejía et al. [17] with minor adjustments. The extracts were analyzed using an Agilent liquid chromatography system (Mod. 1100 Series), outfitted with a G1376A binary capillary pump, a G1379A degasser, a simple quadrupole mass analyzer (6120), and a micro electrospray ionization source (ESI). Agilent Chemstation B.04.01 software was utilized to gather and process data, while an external stainless-steel loop (10 μL) was positioned into a Rheodyne^®^ injection valve. A Synergi™ Fusion C18 capillary analytical column (150 × 0.3 mm i.d., 4 μm, Phenomenex, Torrance, CA, USA) and a mobile phase consisting of 0.05% (*v*/*v*) formic acid aqueous solution at pH 2.9 (A) and acetonitrile (B), operating in gradient elution mode at 10 μL·min^−1^*,* were used to separate the phenolic compounds. The gradient was run as follows: 8% B for 0 to 3 min, 8% to 34% B for 3 to 17 min, 8% to 34% B for 17 to 21 min, and 8% to 34% B for 21 to 24 min. Prior to analysis, each mobile phase was filtered with nylon membrane filters (0.22 m, Teknokroma, Barcelona, Spain). The molecular ion [M − H]^−^ was chosen for online identification of polyphenols using mass detection in negative ion mode, where ESI capillary voltage was fixed at 3.5 kV. The drying gas temperature was set to 325 °C and a flowrate of 8.0 L min^−1^. The nebulizer pressure was fixed at 17 psi. Identification of phenolic compounds was carried out by comparing the retention times and molecular ions acquired from the standards with those of the sample extracts. External calibration curves were obtained for quantifying purposes. For on-column focusing purposes, injection solutions were prepared by adding to sample aliquots 50 μL of ACN, 800 μL of MeOH, 0.05% (*v*/*v*) aqueous formic acid solution at pH 2.9, and Milli-Q water to a final volume of 5 mL.

For a more thorough and wider determination of the bioactive compounds present in the mandarin residues and in the samples subjected to the in vitro digestion process, high-performance liquid chromatography coupled to a quadrupole time-of-flight mass spectrometer (HPLC-ESI-QTOF) was employed. The phenolic analysis was performed in an Agilent liquid chromatography system (Mod. 1200), a quaternary pump (G1311A), a coupled degasser (G1322A), a thermostated automatic injector (G1367B), a thermostated column module (G1316A), and a QTOF mass spectrometer (Agilent G6530A) with atmospheric-pressure electrospray ionization source (ESI) and JetStream technology, operating in negative mode and scanning mode (SCAN) in the *m*/*z* range 100–1000, using a capillary voltage of 4 kV and a 45 psi pressure. Data processing was performed with Masshunter Data Acquisition B.05.00 and Masshunter Qualitative Analysis B.07.00. Nitrogen was used as both nebulizing and drying gas (10.0 L·min^−1^, 325 °C).

HPLC-QTOF separation was performed on a Synergi™ C18 Fusion-RP 80 Å analytical column (150 mm × 3 mm I.D., 4 μm, Phenomenex, USA), maintained at 30 °C and using a mobile phase gradient based on a mixture of 0.1% (*v*/*v*) FA in ACN (solvent A) and 0.1% (*v*/*v*) FA aqueous solution (solvent B) as follows: 10% solvent B holding for 0.1 min, linear increase to 35% B within 30 min, and to 70% B within another 5 min. This condition was held for 2 min, then a final linear increase to 90% B was attained within 3 and was held for 5 min, subsequently followed by a re-equilibration of the column. The flow rate was set at 0.50 mL·min^−1^ and the injection volume was fixed at 20 μL. 

The identification of phenolic compounds was carried out by comparing the retention times and the *m*/*z* of the molecular ions [M − H]^−^ acquired from the standards with those of the sample extracts. For quantification purposes, external calibration curves were obtained. Nevertheless, the effect of the matrix on the suppression/enhancement of the ESI/MS signal was studied by comparing the peak areas of the 2 mg·L^−1^ standard solutions with those obtained for the samples spiked in the former standard mixture. The matrix effect was then calculated using the modified version of the equation described by Matuszewki [11] (Equation (1)).

(1) $$\mathrm{matrix}\;\mathrm{effect},\;\%=\left(\frac{A_{\mathrm{sample}}}{A_{\mathrm{standard}}}\right)\cdot 100$$

The performance of both LC-MS methods was evaluated for the standard solutions under optimal chromatographic conditions in terms of linear range and limits of detection (LOD) and quantification (LOQ) [18,19]. Linear ranges (n ≥ 4) were set at concentrations between 0.002 μg·L^−1^ and 2000 mg·L^−1^. Chromatographic peak areas were analyzed using linear least squares regression, and linearity was assessed as determination coefficients (R^2^). LOD and LOQ were calculated for analyte concentration as 3 signal-to-noise (*S/N*) at height and 10 *S/N*, respectively. Finally, the precision was estimated at 80 μg·L^−1^ for gallic acid, dihydroxybenzoic acid, *p*-coumaric acid, resveratrol, quercetin, and kaempferol; 44 μg·L^−1^ for caffeic acid; 40 μg·L^−1^ for *trans*-ferulic acid; and 480 μg·L^−1^ for catechin. Intraday variation (n = 3) was assessed by injecting three standard solutions at the target concentration for each analyte on the same day, while inter-day precision was similarly obtained from three successive days (n = 9, three injections per day). The relative standard deviation (RSD, %) was calculated for both the retention factor (*k*) and peak areas of each analyte.

### 2.9. In Vitro Gastrointestinal Digestion of Phenolic Compounds from Bread

The 4% (*w*/*v*) fortified and control wheat bread were subjected to an in vitro digestion process, according to the procedure described by Minekus et al. [20], slightly modified. The salivary phase was initiated by mixing 0.2500 g of minced bread with 40 mg of α-amylase and 4 mL of simulated salivary fluid (KCl (179.2 g·L^−1^), KSCN (40 g·L^−1^), NaH_2_PO_4_ (177.6 g·L^−1^), Na_2_SO_4_ (114.0 g·L^−1^), NaCl (350.6 g·L^−1^), NaHCO_3_ (169.4 g·L^−1^), and urea (50.0 g·L^−1^). The pH was set to 6.8 with 0.1 M HCl and incubated at 37 °C for 15 min. Subsequently, 5 mL of pepsin dissolved in 0.1 M HCl was combined, the pH was adjusted to 1.8 with 6 M HCl, and the gastric mixture was maintained at 37 °C for 2 h. The duodenal phase was then initiated by adjusting the pH to 6.8 with saturated aqueous NaHCO_3_ solution. Lastly, 3 mL of 1.5% (*w*/*v*) pancreatin and 0.15% (*w*/*v*) bile salts, both dissolved in 0.15 M NaCl, were added and the mixture was incubated at 37 °C for 2 h. Gastrointestinal digestion blanks were prepared in parallel. The procedure was performed in triplicate with constant shaking (100 rpm) and digested samples were analyzed fresh to prevent phenolic degradation, following the procedure described in Section 2.8.

Finally, the in vitro bioaccessibility index (IVBA), i.e., the percentage content of a polyphenol at the duodenal stage following the in vitro gastrointestinal step [12], was determined, when possible, by applying Equation (2).

(2) $$\mathrm{IVBA},\;\%=\left(\frac{{\left[\mathrm{Polyphenol}\right]}_{\mathrm{duodenal}\;\mathrm{stage}}}{{\left[\mathrm{Polyphenol}\right]}_{\mathrm{initial}\;}}\right)\cdot 100$$

### 2.10. Statistical Analysis

Data were statistically analyzed using two-tailed paired *t*-Student test, one-way analysis of variance (ANOVA), and the least-significant difference (LSD) multiple comparison test using the software package Statgraphics 19 (Statgraphics Technologies Inc., Rockville, MD, USA).

## 3. Results and Discussion

### 3.1. Performance of the Chromatographic Methods

A cLC-ESI-MS analytical method was developed, optimized, and validated for the determination of gallic acid, dihydroxybenzoic acid (DHB), catechin, caffeic acid, *p*-coumaric acid, *trans*-ferulic acid, resveratrol, quercetin, and kaempferol, seeking the optimal chromatographic resolution in the minimum analysis time. Based on the LC separation proposed by Gómez-Mejía et al. [21], the former nine compounds were determined in less than 25 min (Table S1). Calibration was performed using analyte standard solutions, where linearity and correlation coefficients were determined via external calibration using peak area values as responses. As shown in Table S1, wide linear ranges were found, namely for kaempferol, quercetin, resveratrol, and *p*-coumaric acid (0.5–180 μg·L^−1^), with R^2^ values above 0.9900. Further, acceptable LODs and LOQs were estimated in the range 0.1–6.0 μg·L^−1^ and 0.3–20 μg·L^−1^, respectively, apart from catechin (LOD = 100 μg·L^−1^), for which a lower sensitivity was observed, possibly associated with the negative ionization mode [22]. In addition, the repeatability and intermediate precision of the cLC-ESI-MS method were assessed for each polyphenol using the retention factor and peak area. Satisfactory RSD values were observed for all polyphenols analyzed, with RSD values below 7.8%.

To complement this analysis, an HPLC-ESI-QTOF method was also developed and validated to determine a larger number of phenolic compounds, including those reported to be abundant in citrus peels, i.e., hesperidin, naringin, and rutin [15,23]. Exact-mass analysis offered the advantage of unequivocally identifying those bioactive compounds present in the extracts, as well as enhancing selectivity, which is particularly relevant when analyzing complex matrices, such as digested samples. Analyte standard solutions allowed for performing calibration curves (n = 4) by using peak area values, showing wide linear ranges (2–2000 μg·L^−1^) and suitable R^2^ (≥0.9990) (Table S2). On the other hand, appropriate LODs and LOQs were estimated, under 8.9 μg·L^−1^ and 28.7 μg·L^−1^ (Table S2), respectively, evidencing an adequate performance of the method. The matrix effect was evaluated by comparing the peak areas of pure standard with those of spiked samples (undigested and digested samples), as described Equation (1). A marked matrix effect was observed for all the polyphenols studied in the undigested and digested bread samples. In bread extract, this effect was less than 61%, apart from *trans*-ferulic acid, which showed a signal inhibition of 91%. As for the digested samples, the highest matrix effect was observed in the salivary stage given the high salinity of this digestive fluid (above 90% except for quercetin and kaempferol, both of which had 76% inhibition). Furthermore, the gastric phase showed a matrix inhibition below 70% by most of the phenolic compounds, while in the duodenal phase this effect was enhanced, especially for gallic acid, DHB, caffeic acid, and *p*-coumaric acid (>91%). Therefore, the quantification of the samples was carried out considering the inhibition percentages determined with Equation (1).

### 3.2. Determination of the Extraction Conditions of Polyphenols from Wheat Bread

Prior to the evaluation of the functionality of the enriched bread, an initial extraction step of phenolic compounds is required. Thus, to select the most suitable extraction method, different techniques were evaluated, namely, conventional SLE extraction (assisted by mechanical stirring or magnetic stirring with heating) and UAE, using different equipment: an HD ultrasonic homogenizer with a 15.5 mm diameter probe and a Vibra-Cell homogenizer with a 2 mm tip. Conventional solvent extraction was selected because of its widespread use for phenolic extraction in a variety of agri-food matrices, including wheat bread [2]. Furthermore, UAE, a non-conventional technique employed in phenolic extraction from bakery products [24,25], was proposed based on its potential to shorten extraction times, promote matrix tissue breakdown, and increase the mass transfer surface area of the phenolic compounds [26]. On the other hand, all extractions were based on the use of ethanol–water 70:30 (*v*/*v*) mixtures, since they are considered efficient, GRAS, and environmentally friendly [17].

**Figure 1:**
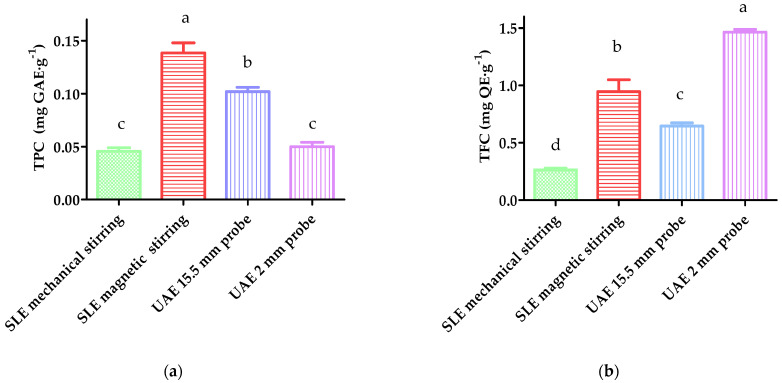

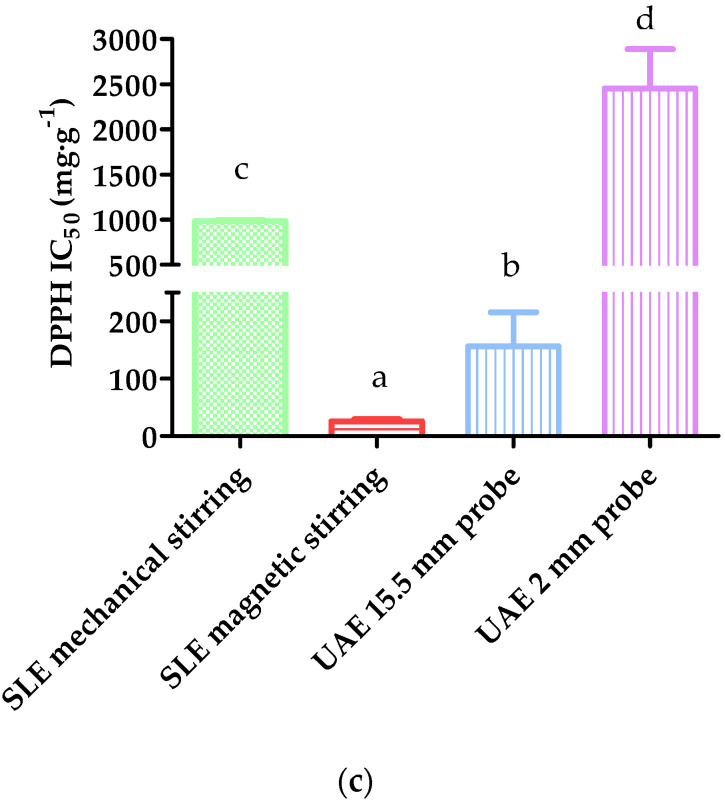
Comparison of the different extraction techniques used in the recovery of phenolic compounds from bread, according to (**a**) total phenolic content; (**b**) total flavonoid content; and (**c**) DPPH antioxidant activity. Data with different letters are significantly different at *p*−value < 0.05, according to one-way ANOVA and Fisher’s LSD test.

The extraction efficiency of each tested condition was expressed in terms of the TPC, TFC, and DPPH antioxidant activity and compared using ANOVA and LSD tests, showing significant differences (*p*-value < 0.05) in all determinations. As can be observed in Figure 1a,c, the SLE assisted by magnetic stirring and heating showed the highest TPC (0.14 mg GAE·g^−1^) and antioxidant activity, i.e., the lowest IC_50_ (28 mg·g^−1^), followed by the UAE performed with the 15.5 mm probe. Positive correlations have been found between the TPC and the antioxidant activity of phenolic extracts [2,3,12]. Particularly, gallic acid, dihydroxybenzoic acid, and ferulic acid have been described to be potent antiradical agents present in wheat, flour, and bakery samples [3,21]. Moreover, in line with the obtained results, other authors have described the better efficiency of conventional solid–liquid extraction versus ultrasound-assisted extraction, mainly aimed at the recovery of hydroxycinnamic acids [27], which can be due to the oxidation of the phenolic compounds by the formation of free radicals [28].

For the TFC, shown in Figure 1b, the SLE assisted by magnetic stirring and heating was significantly lower than the UAE performed with the 2 mm probe (0.9 mg QE·g^−1^ and 1.46 mg QE·g^−1^, respectively). The better results of the probe in this respect could be due to the more efficient release of the matrix-bound flavonoids present in the wheat, such as rutin [29], associated with the massive energy released by the ultrasound probe [28]. Furthermore, this compound is not very efficient against DPPH radicals [21], which in turn would account for the higher IC_50_ value compared with the SLE (Figure 1c).

Overall, given the importance of antioxidant activity in the functionality of bakery products and its good correlation with TPC [3,16], as well as the easiness of handling, SLE assisted by stirring and heating was selected as the most favorable technique for extracting bread polyphenols. These results agree with previous studies reporting a higher efficiency of SLE in the recovery of phenolic compounds compared with UAE [30], which could be related to the lower potential to extract other non-phenolic compounds, such as polysaccharides, which not only impair the selectivity but also increase the viscosity of the extract [31].

### 3.3. Total Contents and Antioxidant Characterization of Mandarin Peel and Enriched Bread

**Figure 2:**
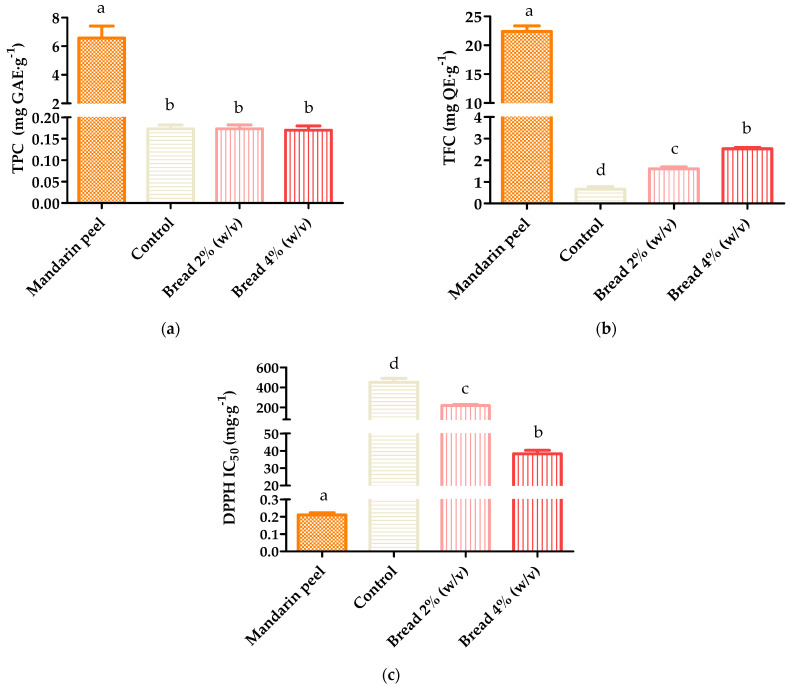
Spectrophotometric determinations of phenolic extracts of mandarin peel, control bread, and enriched bread at 2% (*w*/*v*) and 4%(*w*/*v*): (**a**) total phenolic content; (**b**) total flavonoid content; (**c**) DPPH antioxidant activity. Data with different letters are significantly different at *p*−value < 0.05, according to one-way ANOVA and Fisher’s LSD test.

Citrus extracts were obtained following the SLE procedure described in Section 2.4, given its simplicity, improved efficiency, and easy application on an industrial scale. In this method, a 20:80 (*v*/*v*) ethanol–water mixture was used, due to its greater effectiveness compared with extraction with water alone [15]. In addition, ethanol is a Generally Recognized as Safe solvent according to the American Food and Drug Administration [32], so its use avoids the disposal of the solvent, moving towards zero-waste processes, and harmonizes the applicability of ready-to-use phenolic extract with enhanced functionality in the food industry [11,32]. Analogously, the phenolic extracts of the control and enriched breads were obtained according to the SLE method described in Section 2.6, due to its greater effectiveness in the extraction of compounds with DPPH-radical-inhibition capacity, as previously established. The phenolic extracts obtained were characterized in terms of TPC, TFC, and DPPH antiradical activity (Figure 2), which provided rapid and practical information on the variability of phenolic composition in foods and extracts [12,15], and they were then compared with ANOVA and LSD tests, showing significant differences (*p*-value < 0.05) in all determinations. According to the data included in Figure 2, both total content and antioxidant activity were significantly higher in the mandarin peel extract than in the control and functionalized breads. This is due not only to the fact that citrus peels are particularly abundant in antioxidants [6], but also to the loss of the grain polyphenols in the processing of refined flour and during bread baking [3].

As a basis for comparison, Anticona et al. determined a TPC ranging from 6 to 9 mg GAE·g^−1^ and a TFC varying between 4 and 6 mg CE·g^−1^ when analyzing mandarin peel extracts (*C. reticulata* Blanco × *C. Sinensis* Osbeck) with EAU [33], with the values reported in this study being comparable or even higher (TPC = 7 ± 1 mg·g^−1^ and TFC 22 ± 1 mg·g^−1^), demonstrating their potential as a source of high value-added compounds. Similarly, the antioxidant activity determined in this study was over 63 mg/100 g, exceeding the value reported herein (IC_50_ = 0.95 mg·g^−1^) for a mandarin peel citrus residue. The higher antioxidant activity of the present extract may be related to a greater content in phenolic compounds with significant free-radical-inhibition activity, such as gallic acid and quercetin [21]. Thus, the promising results of this mandarin peel extract could be attributed to both the efficiency of the extraction method and the polyphenolic potential of the residue evaluated, suggesting its prospective for the fortification and functionalization of white bread.

As for the total indices and antioxidant activity of the fortified breads, a significant dependence on the percentage of fortification was observed for both TFC and DPPH, in contrast to TPC, where all values were roughly comparable (Figure 2). The lack of effect of bread fortification on the TPC could be caused by the presence of sugars, especially abundant in white bread [3], in the extract obtained using SLE with an ethanol–water 70:30 (*v*/*v*) mixture. These sugars also react with the Folin–Ciocalteu reagent and may interfere with the determination and thereby alter the outcome, as previously stated [21]. Conversely, Cedola et al. observed that the TFC of *taralli* (Italian baked bread) quadrupled from 0.09 mg QE·g^−1^ to 0.36 mg QE·g^−1^ when the dough was enriched with 17% (*w*/*w*) olive leaf extract [34]. A similar trend was reported by Laganà et al. when they studied cookies enriched with bergamot (*Citrus bergamia*) pomace. In particular, a significant proportional increase in TFC was recorded when the citrus residue replaced from 2.5% to 15% (*w*/*w*) of the wheat flour in the formulation [10]. On the other hand, the improvement in the antioxidant activity of the 4% (*w*/*v*) enriched bread was quite substantial, with an IC_50_ reduction of more than tenfold (IC_50_ = 38 ± 2 mg·g^−1^) compared with the control bread (IC_50_ = 451 ± 40 mg·g^−1^), while the 2% (*w*/*v*) enriched bread halved its IC_50_ over the control (Figure 2c). Accordingly, the antioxidant potency of citrus fortification in baked goods has been observed in other studies [8,9], highlighting that supplementation with extracts is much more profitable due to the higher purity of phytochemicals compared with whole-residue fortifiers [9].

### 3.4. Individual Phenolic Composition of Mandarin Peel and Enriched Bread

While spectrophotometric methods are very useful for estimating total phenolic content and antioxidant activity, their lack of selectivity and information regarding the identity of the polyphenols present in the extracts make it necessary to complement the characterization of phenolic extracts using chromatographic methods [12]. As such, the phenolic extract obtained from mandarin peel, control, and fortified breads were analyzed with cLC-ESI-MS and LC-ESI-MS/MS. In total, ten phenolic compounds were identified and quantified, when possible, in the mandarin peel extract (Table 1). The use of HPLC-ESI-MS/MS enabled the unequivocal identification of those bioactive compounds present in the extract, as well as a selectivity improvement, particularly relevant in the analysis of complex samples, such as bread. Thus, HPLC-ESI-MS/MS analysis confirmed the presence of dihydroxybenzoic acid, *p*-coumaric acid, *trans*-ferulic acid, and quercetin, at concentration levels consistent with those obtained using cLC-ESI-MS. Moreover, it allowed the determination of caffeic acid and kaempferol, given a higher sensitivity and selectivity (Tables S1 and S2). However, gallic acid was not detected at the LOD levels of the HPLC-ESI-MS/MS method, although still quantified using cLC-ESI-MS, possibly due to the enhanced LOD of the latter, as it combines the use of the capillary LC column and the on-column focusing technique, thus increasing the sensitivity for these phenolic acid determinations [12]. As a novelty, naringin, hesperidin, rutin, and myricetin were identified and quantified by means of the HPLC-ESI-MS/MS method in the control and 4% (*w*/*v*) fortified bread (Table 1). These phenolic compounds could not be included in the cLC-ESI-MS method, since, as described before, they present a limited ionization, impairing their MS sensitivity against UV-Vis detectors [16,21]. Therefore, for comparative and unequivocal identification purposes, only the phenolic extracts from mandarin peel, the control bread, and the 4% enriched bread were analyzed using HPLC-MS/MS.

Table 1 summarizes the phenolic compounds determined in the phenolic extracts of mandarin peels and the studied breads. The mandarin peel extract was found to be significantly most abundant in the flavonoid family, mainly due to the richness of hesperidin (6.2 mg·g^−1^). Several studies have reported the mentioned flavanone as the predominant phenolic compound in citrus peels, as such hesperidin has been determined in concentrations ranging from 1 to 15 mg·g^−1^ in *Citrus reticulata* Blanco [35,36] and *Citrus* × *clementina* peels [5,16]. These results agree with our data, pointing out that variability in concentration is most likely attributable to the extraction method, the genotype, and variety of the mandarin fruit or the environmental conditions in which it grew [4]. Moreover, the flavone myricetin was found at a significant concentration, 138 µg·g^−1^, followed by quercetin and kaempferol, which were quantified at levels around 60 µg·g^−1^, and, lastly, by rutin and naringin, which were minor components (Table 1). Closely, other authors have found myricetin as a rich compound in *Citrus sinensis* peels [37], while quercetin, kaempferol, and naringin were determined at low concentrations in *Citrus reticulate* L. [36] and *Citrus* × *clementina* peel extracts [15], respectively. As for rutin, a great variability in its concentration was observed, fluctuating from not detected to 13 mg·g^−1^, in different mandarin peel extracts [36], again probably due to the extraction condition and the environmental and genetic factors [4]. As regards phenolic acids, these accounted for 7% of the total phenolic composition, with gallic acid standing out with a concentration of 280 µg·g^−1^, followed by *trans*-ferulic, *p*-coumaric, and caffeic acid, which were present at levels between 96 and 38 µg·g^−1^ (Table 1). Consistent with the results obtained, a low amount of caffeic acid (0.32–1.6 mg/g) was also found by Safdar et al. [36]; in contrast, gallic, *p*-coumaric, and *trans*-ferulic acids have been described as the leading acids in peel extracts of *Citrus reticulata* Blanco [38] and *Citrus* × *clementina* [16]. As for the control bread, more than 70% of the total polyphenol content corresponded to phenolic acids, specifically hydroxycinnamic acids, namely *trans*-ferulic acid (2.01 ± 0.03 µg·g^−1^) and *p*-coumaric acid (3.50 ± 0.07 µg·g^−1^); followed by the hydroxybenzoic acids; gallic acid (3.52 ± 0.01 µg·g^−1^); and dihydroxybenzoic acid, whose concentration did not exceed 1 µg·g^−1^ (Table 1). Hesperidin, rutin, and resveratrol appeared in a concentration range between 0.5 and 2 µg·g^−1^. Coherent with the above results, phenolic acids have been reported to be more abundant than flavonoids in wheat, flour, and bakery products [3], as well as in extra virgin olive oil [39]. Ferulic acid accounted for more than 59% of the total phenolic acids in refined bread and whole bread, observing *p*-coumaric acid concentrations of 4.5 µg·g^−1^ [40]. On the other hand, Sharma et al. [29], reported that different wheat varieties contained concentrations of hesperidin and rutin (3 µg·100 g^−1^) comparable to those in this study (Table 1). It is also interesting to consider the contribution of extra virgin olive oil to the phenolic composition of prepared bread, although to a lesser extent than wheat, since it accounts for 3% of the bread dough. In this sense, the presence of 3,4-dihydroxybenzoic acid, *p*-coumaric acid, *trans*-ferulic acid, and quercetin has been described in extra virgin olive oil marketed at concentrations lower than 0.46 µg·g^−1^, while kaempferol was not detected [39], consistent with the results reported in Table 1. Overall, mandarin peel extracts were found to be more plentiful in phenolic compounds, with a total amount of 7 mg·g^−1^ of the polyphenols screened vs. 12.9 ± 0.3 µg·g^−1^ in the control bread, demonstrating their potential as a source of bioactive polyphenols to be used as functional ingredients in the development of enriched bread.

As for the effect of bread enrichment, Table 1 shows significant differences in the profile and content of the fortified breads, both with the control bread and with the crude mandarin peel extract. As for the sum of individual polyphenols, it was three times higher in the bread enriched at 2% (*w*/*v*) compared with the control bread, while in the bread enriched at 4% (*w*/*v*), it was twelvefold higher (Table 1). These results clearly indicate the potential of the 4% (*w*/*v*) enriched bread to improve the total polyphenol content of wheat-based baking products. Specifically, the main effect of mandarin peel phenolic extract fortification was a significant increase in the flavonoid fraction, mainly focused on quercetin, myricetin, and naringin, which were not previously found in the control bread (Table 1), but also on hesperidin, whose concentration reached 83 µg·g^−1^ in the 4% (*w*/*v*) enriched bread and was the main phenolic compound in mandarin peel (Table 1). Likewise, the addition of mandarin peel extract to the dough had a positive effect on the content of *trans*-ferulic acid and gallic acid, with comparable concentrations at both levels of enrichment (*p*-value > 0.05), probably due to the bonding of their free forms to the matrix components because of the baking process [3]. On the contrary, no effect was observed for kaempferol, which was not detected in either of the two enriched breads, nor when it came to *p*-coumaric acid, where a lower concentration (*p*-value < 0.05) was observed for the enriched bread compared with the control. Likewise, Xiao et al. reported the loss of free kaempferol aglycone in a fermented buckwheat bread dough after steaming [41]. These forfeits could be attributed to the oxidation of kaempferol to the benzofuranone form, favored not only by the temperature and heat used during baking, but also by the presence of oxidative enzymes such as polyphenol oxidase, which also leads to the development of the characteristic brown color of the bread [42]. On the other hand, the lower presence of free *p*-coumaric acid in the enriched breads compared with the control could be attributed to the role of this polyphenol in the inhibition of acrylamide, according to the well-known Millard reaction, which is favored in the presence of reducing sugars, which are possibly co-extruded in the mandarin extract [15,42,43]. Xu and An revealed that *p*-coumaric acid most likely reacted with a precursor of acrylamide (3-oxopropanamide), giving rise to 2-(propenamide)coumaric acid [42]. Resveratrol, which was not identified in the mandarin peel extract, showed no significant differences between the breads (Table 1), while dihydroxybenzoic acid was significantly increased in the 2%(*w*/*v*) and 4%(*w*/*v*) enriched bread, with a concentration around 40 µg·g^−1^ in each. This could be related to the hydrolysis of the higher-molecular-weight dihydroxybenzoic acid derivatives, since the phenolic extract would have a lower pH than the EtOH–water mixture used in the control bread preparation, thus supporting their breakdown [44]. Similarly, Taglieri et al., concluded that some phenolic compounds, such as feruloyl acid, diosmetin, and glucosidase eriocitrin, were lost during the preparation of bread enriched with 0.75% (*w*/*w*) sour orange albedo, compared with the control [9]. Therefore, the addition of phenolic compounds in food matrices does not always translate to a final enrichment, whereby the effect of the cooking and the matrix plays a pivotal role in the final composition [3].

### 3.5. In Vitro Gastrointestinal Digestion Study of Phenolic Compounds in Bread

Determining the content of phenolic compounds in foodstuff is not sufficient to predict their potential bioactivity. Indeed, it is well-known that the most widespread polyphenols in foods and beverages are not necessarily the most accessible, nor the most active, given that to perform their function, polyphenols must be released from the matrix, transferred into the bloodstream, and reach up to the target tissue or organ while undergoing mechanical, enzymatic, and metabolic transformations [13]. Hence, a first approach to investigate the effect of the digestive environment on the phenolic stability is to submit the foodstuff to an in vitro gastrointestinal assay [13,34]. In this study, fifteen monomeric and low-molecular-weight phenolic compounds were carefully selected, including phenolic acids, flavonoids, and stilbenes, since these properties would favor their bioaccessibility and eventual bioavailability [12]. In addition, the in vitro digestive stability study of the polyphenols evaluated was performed using the bread itself (control and enriched) and not the derived extracts, since the presence of other phytochemicals and the matrix is essential to representatively assess the bioaccessibility of phenolic compounds in any food [12,13]. As can be seen in Table 2, seven phenolic compounds were identified and quantified, when possible, in all or some of the digestive stages, namely the phenolic acids dihydroxybenzoic, caffeic, *p*-coumaric, and *trans*-ferulic acids and the flavonoids rutin, hesperidin, and quercetin. 

Considering the salivary stage, the total polyphenol content was significantly higher in the 4% (*w*/*v*) enriched bread (7.1 ± 0.3 µg·g^−1^) compared with the control (1.71 ± 0.05 µg·g^−1^), demonstrating the effectiveness of fortification. *p*-Coumaric acid and rutin were determined in comparable concentrations in the salivary phases of both breads (Table 2), while *trans*-ferulic acid was higher in the control bread, and hesperidin, quercetin, and caffeic acid were only determined in the salivary extract of the 4% (*w*/*v*) enriched bread, as these compounds came directly from the mandarin extract (Table 1). Several authors have described good stability of polyphenols such as quercetin, caffeic acid, and *trans*-ferulic acid under oral biochemical conditions [12,45,46].

However, this condition is greatly altered under gastric environments, observing a general shift in both the content and distribution of polyphenols in the bread (Table 2). In this context, a total loss of the previously determined phenolic acids (*p*-coumaric, *trans*-ferulic, and caffeic), as well as of the flavonol quercetin, occurs in both bread samples, either due to acid hydrolysis caused by the low pH of the gastric medium or by their interaction with pepsin via non-covalent bonds [12,46]. On the contrary, dihydroxybenzoic acid appears for the first time at this stage in both samples and at a similar concentration (around 0.08 µg·g^−1^) (Table 2). This fact could be due to the release of dihydroxybenzoic acid bound to the baking matrices in acidic media. Previously, Arranz et al. described this behavior when studying the extraction of dihydroxybenzoic acid in wheat flour, inferring that an appreciable number of polyphenols bound to cell-wall constituents or trapped within the food matrix may remain insoluble after aqueous–organic extraction and/or alkaline treatment [44]. Rutin and hesperidin were also released in the gastric phase, with a significantly higher concentration in the 4% (*w*/*v*) enriched bread (Table 2). In agreement with current findings, some authors have noted a rather remarkable gastric stability of flavonoid glycosides, such as rutin and hesperidin, even reporting increases in their concentration. As such, it has been suggested that the cleavage of polyphenols with a higher degree of glycosylation in the gastric environment could lead to those disaccharide flavonoids of lower molecular mass [12,47].

After the duodenal phase, complete depletion of phenolic compounds occurs, except for hesperidin (Table 2). A similar observation was previously made by Cedola et al. when studying the digestive stability of oleuropein, hydroxytyrosol, and verbascoside in Italian breads enriched with 17% (*w*/*w*) olive leaf extract, reporting such a significant decrease that the enrichment effect was suppressed [34]. Consequently, it can be safely stated that the degradation of bioactive compounds occurs mainly in the intestine, where a mild alkaline pH facilitates the epimerization and auto-oxidation of these compounds in addition to the interaction of phenolic compounds with pancreatin, which reduces the phenolic free form [34,45,46]. As regards hesperidin, it highlights the progressive enrichment of this flavonoid over the different digestive stages (Table 2), which could be explained by the release of matrix-bound hesperidin and/or deglycosylation of high-molecular-weight glycoside derivatives, particularly favored at the mild alkaline pH of the duodenum, as previously reported during the digestion of white tea and whole grapes [12,48]. Hesperidin presented in vitro bioaccessibility indexes above 100% in both cases, reaching (220 ± 6)% in the 4% (*w*/*v*) enriched bread, with double the duodenal concentration in the aforementioned case ((159 ± 36) µg·g^−1^ vs. (77 ± 5) µg·g^−1^ in the control). This fact is noteworthy, not only because it demonstrates the relevance and potential of mandarin-peel-enriched bakery products, but also because different models have shown that hesperidin consumption prevents lung damage, modulates antioxidant enzymes that induce apoptosis and cancer cell proliferation, and prevents cognitive deficits in cell lines, mice, and even in humans [49]. This suggests that consumption of the fortified bread proposed in this study could have beneficial health impacts in consumers, although further in vivo and clinical studies should be conducted.

## 4. Conclusions

This study has proposed the valorization of citrus peel wastes by obtaining an extract rich in phenolic compounds, which was subsequently used in the preparation of an enriched bread. In particular, the effect of the enrichment percentage of phenolic extract has been evaluated in terms of antioxidant activity and phenolic content. A significant increase in the DPPH free-radical-inhibition potential and in the content of total flavonoids, in particular hesperidin, myricetin, and quercetin, has been observed to a greater extent in the bread enriched at 4% (*w*/*v*). In addition, a study of the digestive stability of the polyphenols in the 4% (*w*/*v*) enriched bread was carried out and compared with the control bread. The results obtained showed a higher content of total individual polyphenols in the functionalized bread at all digestive stages, although, due to changes in the pH of the medium and digestive enzymes, only hesperidin could be quantified in the duodenal stage, with a concentration as high as 150 µg·g^−1^. Thus, such utilization of mandarin peels opens the way to an ecological and low-cost alternative to synthetic additives, with the aim of producing enriched foodstuff with potential health benefits for consumers.

## Figures and Tables

**Table 1:** Phenolic compounds determined in mandarin peel, control, and fortified wheat bread extracts. Data are expressed as mean ± standard deviation (n = 3).

Compound (µg·g^−1^ DW)	Mandarin Peel	Control Bread	2% (*w*/*v*) Fortified Bread	4% (*w*/*v*) Fortified Bread
Gallic acid	280 ± 6 ^a^	3.52 ± 0.01 ^c^	5.3 ± 0.5 ^b^	5.6 ± 0.5 ^b^
Dihydroxybenzoic acid	*n.d.*	0.29 ± 0.02 ^b^	0.42 ± 0.03 ^a^	0.44 ± 0.02 ^a^
Caffeic acid	38 ± 1 ^a^	*n.d.*	*n.d.*	0.75 ± 0.02 ^b^
*p*-Coumaric acid	72.4 ± 0.2 ^a^	3.50 ± 0.07 ^b^	2.28 ± 0.07 ^c^	2.34 ± 0.09 ^c^
*trans*-Ferulic acid	96.2 ± 0.5 ^a^	2.01 ± 0.03 ^c^	2.4 ± 0.2 ^bc^	2.57 ± 0.08 ^b^
Rutin	45 ± 4 ^a^	2.06 ± 0.01 ^c^	*n.a.*	7.3 ± 0.5 ^b^
Naringin	13 ± 1 ^a^	*n.d.*	*n.a.*	3.0 ± 0.2 ^b^
Hesperidin	(6.2 ± 0.9) × 10^3 a^	1.1 ± 0.4 ^c^	*n.a.*	83 ± 3 ^b^
Myricetin	138 ± 20 ^a^	*n.d.*	*n.a.*	16.6 ± 0.9 ^b^
Resveratrol	*n.d.*	0.502 ± 0.001 ^a^	0.501 ± 0.001 ^a^	0.502 ± 0.002 ^a^
Quercetin	63 ± 3 ^a^	*n.d.*	22.8 ± 0.6 ^c^	33.3 ± 0.6 ^b^
Kaempferol	56 ± 8	*n.d.*	*n.d.*	*n.d.*
Total Phenolic Acids	489 ± 8 ^a^	9.3 ± 0.1 ^b^	10.7 ± 0.03 ^b^	12.4 ± 0.3 ^b^
Total Flavonoids	6400 ± 900 ^a^/ *119 ± 10 ^a^*	3.1 ± 0.4 ^d^/ *0.502 ± 0.01 ^c^*	*22.7 ± 0.2* ^c^	141 ± 3 ^b^/ *33.8 ± 0.2 ^b^*
Total Polyphenols	6900 ± 940 ^a^/ *607 ± 18 ^a^*	12.9 ± 0.3 ^d^/ *9.8 ± 0.1 ^c^*	*33.8 ± 0.2 ^c^*	154 ± 3 ^b^/ *46.3 ± 0.9 ^b^*

Mean values with different letters in the same column indicate significant differences (*p*-value < 0.05) according to ANOVA and Fisher LSD test. *n.d.* = not detected; *n.a.* = not analyzed. Total values in italics exclude hesperidin, myricetin, rutin, and naringin. DW: dry weight (moisture content of: mandarin peels (38.8 ± 0.6)%, control bread (33.8 ± 0.3)%, 2% (*w*/*v*) enriched bread (29.4 ± 0.5)%, and 4% (*w*/*v*) enriched bread (28.8 ± 0.4)%).

**Table 2:** Phenolic compounds determined during the in vitro digestion process using HPLC-ESI-MS/MS in control and 4% (*w*/*v*) enriched bread (mean ± SD, n = 3).

Compound (µg·g^−1^ DW)	Control Bread			4% (*w*/*v*) Fortified Bread		
	Salivary Phase	Gastric Phase	Duodenal Phase	Salivary Phase	Gastric Phase	Duodenal Phase
Dihydroxybenzoic acid	*n.d.*	0.062 ± 0.009	*n.d.*	*n.d.*	0.09 ± 0.01	*n.d.*
Caffeic acid	*n.d.*	*n.d.*	*n.d.*	0.63 ± 0.02	*n.d.*	*n.d.*
*p*-Coumaric acid	1.06 ± 0.07	*n.d.*	*n.d.*	0.979 ± 0.002	*n.d.*	*n.d.*
*trans*-Ferulic acid	0.31 ± 0.06 **	*n.d.*	*n.d.*	0.03 ± 0.01	*n.d.*	*n.d.*
Rutin	0.34 ± 0.03 ^b^	1.57 ± 0.04 ^a^	*n.d.*	0.41 ± 0.02 ^b^	2.6 ± 0.1 ^a^	*n.d.*
Hesperidin	*n.d.*	*n.d.*	77 ± 5 **	3.8 ± 0.2 ^c^	28.7 ± 0.2 ^b^	159 ± 36 ^a^
Quercetin	*n.d.*	*n.d.*	*n.d.*	1.25 ± 0.02	*n.d.*	*n.d.*
Total Phenolic Acids	1.37 ± 0.01 ^a^	0.062 ± 0.009 ^b^	-	2.26 ± 0.06 ^a^	0.09 ± 0.01 ^b^	-
Total Flavonoids	0.34 ± 0.03 ^c^	1.57 ± 0.04 ^b^	77 ± 5 ^a^	5.48 ± 0.20 ^c^	31.3 ± 0.2 ^b^	159 ± 36 ^a^
Total Polyphenols	1.71 ± 0.05 ^b^	1.64 ± 0.03 ^b^	77 ± 5 ^a^	7.1 ± 0.3 ^c^	31.4 ± 0.1 ^b^	159 ± 36 ^a^

Mean values with different letters in the same column indicate significant differences (*p*-value < 0.05), according to ANOVA and Fisher LSD test. ** Statistical differences < 0.05 by applying a two-tailed paired *t*-Student test to different bread samples at the same digestive stage. *n.d.* = not detected. DW: dry weight (moisture content of: mandarin peels (38.8 ± 0.6)%, control bread (33.8 ± 0.3)%, 2% (*w*/*v*) enriched bread (29.4 ± 0.5)%, and 4% (*w*/*v*) enriched bread (28.8 ± 0.4)%).

## Data Availability

All data are contained within the article and Supplementary Material.

## Supplementary Materials

The following supporting information can be downloaded at: https://www.mdpi.com/article/10.3390/antiox12091742/s1, Table S1: Analytical parameters, retention time, and mass spectral data for the identification and quantification of phenolic compounds using the cLC-ESI-MS method, Table S2: Analytical parameters, retention time, molecular formula, and mass spectral data for the identification and quantification of phenolic compounds using the HPLC-ESI-QTOF method.
