# Supplementary material for: Valorization of Citrus reticulata Blanco Peels to Produce Enriched Wheat Bread: Phenolic Bioaccessibility and Antioxidant Potential
Source: Antioxidants (Basel). 2023 Sep 8;12(9):1742. doi: 10.3390/antiox12091742 (PMC10525822; doi:10.3390/antiox12091742)
Supplement: Supplementary file 1 [file antioxidants-12-01742-s001.zip › antioxidants-2556174-supplementary.pdf]

Table S1. Analytical parameters, retention time and mass spectral data for the identification and quantification of phenolic compounds using cLC-ESI-MS method.

Compound	Retention time, min	Precursor Ion [M-H]⁻ (*m/z*) (*Fragmentor, eV*)	Linear range, µg·L^{-1} (n)	*LOD,* µg·L^{-1}	*LOQ,* µg·L^{-1}	Calibration equation, y = *ax* + *b*			Intra-day precision RSD, % (n=3)		Inter-day precision RSD, % (N=9)	
						a, L·µg^{-1}	*b*	R^2	Area	*k*	Area	*k*
**Gallic acid**	3.6	169.0 (*70*)	10-250 (6)	2.0	7.0	(5.7 ± 0.1) · 10^2	(0 ± 1) · 10^3	0.9988	4.0	4.8	5.9	4.3
**DHB**[1]	4.9	153.0 (*70*)	20-250 (5)	6.0	20	(8.5 ± 0.3) · 10^2	(0 ± 4) · 10^3	0.9971	5.7	3.0	6.1	5.4
**Catechin**	9.9	289.1 (150)	240-600 (5)	100	333	(4.7 ± 0.3) · 10^2	(0 ± 1) · 10^4	0.9905	3.4	2.2	4.3	6.0
**Caffeic acid**	11.8	179.0 (*150*)	16-80 (5)	4.0	13	(1.76 ± 0.08) · 10^3	(0 ± 4) · 10^3	0.9932	5.1	1.0	3.9	2.7
***p*-Coumaric acid**	14.5	163.0 (*70*)	2-180 (8)	0.5	1.7	(2.30 ± 0.05) · 10^3	(0 ± 4) · 10^3	0.9973	1.6	0.4	3.0	1.4
***trans*-Ferulic acid**	15.4	193.2 (*70*)	16-80 (5)	4.0	13	(2.05 ± 0.07) · 10^3	(0 ± 4) · 10^3	0.9961	6.1	0.3	7.8	0.7
**Resveratrol**	18.5	227.1 (*150*)	1-150 (8)	0.3	1.0	(7.3 ± 0.2) · 10^2	(0 ± 1) · 10^3	0.9969	6.3	0.3	4.8	0.7
**Quercetin**	19.7	301.0 (*150*)	1-150 (8)	0.3	1.0	(7.4 ± 0.2) · 10^2	(0 ± 1) · 10^3	0.9977	3.6	0.3	5.4	1.2
**Kaempferol**	22.7	285.0 (*150*)	0.5-150 (9)	0.1	0.3	(1.55 ± 0.05) · 10^3	(9 ± 3) · 10^3	0.9931	4.4	0.4	4.2	1.0

[1]DHB: Dihydroxybenzoic acid

Table S2. Analytical parameters, retention time, molecular formula and mass spectral data for the identification and quantification of phenolic compounds using HPLC-ESI-QTOF method.

Compound	Retention time (min)	Molecular formula	Precursor Ion [M-H]⁻ (*m*/*z*) (*Fragmentor, 125 eV*)	Linear range, μg·L^{-1}	*LOD,* μg·L^{-1}	*LOQ,* μg·L^{-1}	Calibration equation, y = *ax* + *b*		
							a, L·μg^{-1}	*b*	R^2
**Gallic acid**	2.9	$C_7H_6O_5$	169.0142	20-2000	8.6	28.7	(1.7 ± 0.1) · 10^7	(1 ± 1) · 10^6	0.9962
**Dihydroxybenzoic acid**	5.0	$C_7H_6O_4$	153.0557	2-2000	1.1	3.7	(1.859 ± 0.004) ·10^7	(0 ± 40) ·10^3	0.9999
**Catechin**	7.5	$C_{15}H_{14}O_6$	289.0718	2-2000	0.9	3.0	(1.62 ± 0.01) ·10^7	(0 ± 13) ·10^3	0.9999
**Caffeic acid**	9.4	$C_9H_8O_4$	179.0350	2-2000	2.5	8.3	(2.98 ± 0.07) ·10^7	(8 ± 7) ·10^5	0.9990
**Epicatequina**	9.6	$C_{15}H_{14}O_6$	289.0718	2-2000	0.8	2.7	(1.31 ± 0.02) ·10^7	(2 ± 2) ·10^5	0.9996
***p*-Coumaric acid**	13.5	$C_9H_8O_3$	163.0401	2-2000	1.1	3.7	(1.70 ± 0.07) ·10^7	(8 ± 7) ·10^5	0.9964
**Rutin**	14.7	$C_{27}H_{30}O_{16}$	609.1461	2-2000	2.0	6.7	(2.23 ± 0.02) ·10^7	(0 ± 2) ·10^5	0.9998
***trans*-Ferulic acid**	14,9	$C_{10}H_{10}O_4$	193.0506	2-2000	1.7	5.7	(5.4 ± 0.1) ·10^6	(1 ± 1) ·10^5	0.9993
**Naringin**	17.4	$C_{27}H_{32}O_{14}$	579.1719	2-2000	0.8	2.7	(1.28 ± 0.02) ·10^7	(2 ± 2) ·10^5	0.9997
**Hesperidin**	17.8	$C_{28}H_{34}O_{15}$	609.1825	2-2000	0.3	1.0	(1.38 ± 0.02) ·10^7	(3 ± 2) ·10^5	0.9997
**Myricetin**	20.6	$C_{15}H_{10}O_8$	317.0303	2-2000	1.7	5.7	(3.56 ± 0.03) ·10^7	(0 ± 3) ·10^5	0.9999
**Resveratrol**	23.0	$C_{14}H_{12}O_3$	227.0714	2-2000	1.1	3.7	(8.18 ± 0.05) ·10^7	(5 ± 5) ·10^4	0.9999
**Quercetin**	26.2	$C_{15}H_{10}O_7$	301.0354	2-2000	0.9	3.0	(3.0 ± 0.1) ·10^7	(1 ± 1) ·10^6	0.9962
**Kaempferol**	31.6	$C_{15}H_{10}O_6$	285.0405	2-2000	1.4	4.7	(3.6 ± 0.1) ·10^7	(0 ± 2) ·10^6	0.9965
